# Structure-adaptive canonical correlation analysis for microbiome multi-omics data 

**DOI:** 10.3389/fgene.2024.1489694

**Published:** 2024-11-20

**Authors:** Linsui Deng, Yanlin Tang, Xianyang Zhang, Jun Chen

**Affiliations:** ^1^ School of Data Science, The Chinese University of Hong Kong, Shenzhen, China; ^2^ School of Statistics, East China Normal University, Shanghai, China; ^3^ Department of Statistics, Texas A&M University, College Station, TX, United States; ^4^ Department of Quantitative Health Sciences, Mayo Clinic, Rochester, MN, United States

**Keywords:** canonical correlation analysis, compositional effect, phylogenetic tree, structural information, dimension reduction, variable selection

## Abstract

Sparse canonical correlation analysis (sCCA) has been a useful approach for integrating different high-dimensional datasets by finding a subset of correlated features that explain the most correlation in the data. In the context of microbiome studies, investigators are always interested in knowing how the microbiome interacts with the host at different molecular levels such as genome, methylol, transcriptome, metabolome and proteome. sCCA provides a simple approach for exploiting the correlation structure among multiple omics data and finding a set of correlated omics features, which could contribute to understanding the host-microbiome interaction. However, existing sCCA methods do not address compositionality, and its application to microbiome data is thus not optimal. This paper proposes a new sCCA framework for integrating microbiome data with other high-dimensional omics data, accounting for the compositional nature of microbiome sequencing data. It also allows integrating prior structure information such as the grouping structure among bacterial taxa by imposing a “soft” constraint on the coefficients through varying penalization strength. As a result, the method provides significant improvement when the structure is informative while maintaining robustness against a misspecified structure. Through extensive simulation studies and real data analysis, we demonstrate the superiority of the proposed framework over the state-of-the-art approaches.

## 1 Introduction

The human microbiome is the collection of microorganisms and their genetic makeup associated with the human body. It plays a critical role in human health and disease ranging from gastrointestinal diseases to various cancers (Sepich-Poore et al., 2021). To gain more mechanistic insights, multi-omics approaches have been increasingly employed in microbiome studies to elucidate the intricate interplay between the environment, the human microbiome and the host at different molecular levels (Hasin et al., 2017; Lloyd-Price et al., 2019). Although many multi-omics datasets have been generated in the past few years, it is unclear how to integrate them efficiently. One useful tool for multi-omics data integration is to perform canonical correlation analysis (CCA). CCA, due to Hotelling (1936), connects two sets of variables by finding a linear combination of variables that maximally correlate. However, the standard CCA fails when the sample size is strictly less than the number of variables as one can find meaningless solutions with correlations equal to one. Also, it does not perform variable selection and hence lacks interpretability. To circumvent these problems, sparse CCA (sCCA) has been proposed, aiming to find pairs of sparse canonical directions by imposing sparsity penalty. The first sCCA algorithm was presented by Parkhomenko et al. (2007), which, however, lacks exact criterion and biconvexity. Witten et al. (2009) applied the penalized matrix decomposition to cross-product matrix and yielded a straightforward formulation for sCCA. Some closely related methods include Parkhomenko et al., 2009; Lê Cao et al., 2009. Hardoon and Shawe-Taylor (2011) expressed the sCCA model as a primal-dual Rayleigh quotient, which takes the primal representation and kernel representation as the first view and second view, respectively. Chu et al. (2013) reformed CCA into a trace maximization problem and computed the sparse solution by the linearized Bregman method. To exploit the potential structural information among features, various forms of structure-adaptive sCCA have been proposed (Lin et al., 2013; Chen et al., 2012; Mohammadi-Nejad et al., 2017). In particular, Chen et al. (2013) proposed the structure-constrained sCCA (ssCCA) to exploit the phylogenetic structure in microbiome data.

Advances in next-generation sequencing technologies have enabled the direct sequencing of microbial DNA to determine microbiome composition, using either targeted or shotgun approaches (Wensel et al., 2022). The resulting microbiome data is typically in the form of a count table that records the frequencies of detected taxa in specific samples. However, due to the complexities inherent in the sequencing process, the total count for a sample reflects the sequencing effort rather than the actual microbial load at the sampling site. Consequently, microbiome data are inherently compositional, meaning that we only have information about the relative abundances of taxa. This compositionality presents significant challenges in the statistical analysis of microbiome data. A change in the (absolute) abundance of one taxon can lead to apparent changes in the relative abundances of all other taxa, complicating the identification of the actual causal taxa (Yang and Chen, 2022). The compositional nature also renders many standard multivariate statistical models inappropriate or inapplicable (Aitchison, 1982). Many efforts have been made to address the compositionality in different contexts of microbiome data analysis. For example, Friedman and Alm (2012) developed an iterative procedure named SparCC that allows inference of correlations for compositional data by assuming that the number of taxa is large and the true correlation network is sparse. Lin et al. (2014) dealt with the variable selection in regression with compositional covariates. Jiang et al. (2019) addressed zero inflation and detected pairs of associated compositional and non-compositional covariates using a Bayesian zero-inflated negative binomial regression model. However, existing CCA methods including ssCCA could not address the compositional effects, potentially reducing its precision in recovering relevant taxa.

We propose a new sCCA framework for integrating microbiome data with other high-dimensional omics data. The framework specifically addresses the compositional nature of the microbiome data. It also allows integrating prior structure information by imposing a “soft” constraint on the coefficients through varying penalization strength. As a result, the method provides significant improvement when the structure is informative while maintaining robustness against a misspecified structure. The developed tool aims to be an important resource for investigators to understand the interplay between the microbiome and host, decipher the molecular mechanisms underlying microbiome-disease association, and identify potential microbial targets for intervention.

This paper is organized as follows. Section 2 introduces the new sCCA framework for integrating microbiome compositional data with (non-)compositional high-dimensional data. Section 3 extends the new framework to incorporate additional prior structural information. In Section 4, we conduct numerical simulations to demonstrate the effectiveness of our proposed methods. Section 5 applies the proposed methods in a real microbiome study to investigate the association between gut bacteria and its metabolic output. We conclude with a discussion in Section 6.

## 2 Compositional sCCA

### 2.1 Formulation

Let us consider two random vectors $X={\left(X_{1},\ldots ,X_{p}\right)}^{⊤}$ and $Y={\left(Y_{1},\ldots ,Y_{q}\right)}^{⊤}$ , where X contains the composition of p taxa and Y is a q -dimensional vector of non-compositional covariates. The nature of the composition makes X lie in a (p−1) -dimensional positive simplex. To address the compositionality, Aitchison and Bacon-Shone (1984) proposed applying the log-ratio transformation to compositional covariates resulting in $Z_{/p}=\left(\log\left(X_{1}/X_{p}\right),\ldots ,\log\left(X_{p-1}/X_{p}\right)\right)$ , where $X_{p}$ is chosen as the reference component. CCA for compositional data can be formulated to find canonical coefficients $a={\left(a_{1},\ldots ,a_{q}\right)}^{⊤}$ and $b_{-p}={\left(b_{1},\ldots ,b_{p-1}\right)}^{⊤}$ so that the correlation between $a^{⊤}Y$ and $b_{-p}^{⊤}Z_{/p}$ is maximized. Note that $b_{-p}^{⊤}Z_{/p}=\sum_{j=1}^{p-1}b_{j}\log\left(X_{j}/X_{p}\right)=\sum_{j=1}^{p}b_{j}\log\left(X_{j}\right)$ with $b_{p}=-\sum_{j=1}^{p-1}b_{j}$ . To avoid the choice of a reference component, we can write the term $b_{-p}^{⊤}Z_{/p}$ in a symmetric form by noticing that $b_{-p}^{⊤}Z_{/p}=b^{⊤}Z$ , where $Z=\left(\log\left(X_{1}\right),\ldots ,\log\left(X_{p}\right)\right)$ and $b=\left(b_{1},\ldots ,b_{p}\right)$ with

$$b\in B_{p}≔\left\{c={\left(c_{1},\ldots ,c_{p}\right)}^{⊤}:\overset{p}{\underset{j=1}{\sum }}c_{j}=0\right\}.$$

Therefore, the compositional CCA aims to find $\tilde{a}$ and $\tilde{b}$ such that

$$\left(\tilde{a},\tilde{b}\right)=\underset{a\in R^{q},b\in B_{p}}{\text{argmax}}\text{Corr}\left(a^{⊤}Y,b^{⊤}Z\right)=\underset{a\in R^{q},b\in B_{p}}{\text{argmax}}\frac{\text{Cov}\left(a^{⊤}Y,b^{⊤}Z\right)}{\sqrt{\text{Var}\left(a^{⊤}Y\right)\text{var}\left(b^{⊤}Z\right)}}.$$

When the dimensions p and q are high (as compared to the sample size), regularization is required to encourage sparsity and to obtain a unique solution to the optimization problem. We let $\Sigma_{YZ}=\text{Cov}\left(Y,Z\right)$ , $\Sigma_{Y}=\text{Cov}\left(Y,Y\right)$ and $\Sigma_{Z}=\text{Cov}\left(Z,Z\right)$ . Define the weighted $l_{1}$ norm based on a vector of non-negative weights $w=\left(w_{1},\ldots ,w_{p}\right)$ for a vector b as $\left\|b{\|}_{1,w}=\sum_{j=1}^{p}w_{j}|b_{j}\right|$ . The compositional sCCA problem can then be formulated as

$$\underset{a\in R^{q},b\in R^{p}}{\max}a^{⊤}\Sigma_{YZ}b\;\text{s.t.}\;a^{⊤}\Sigma_{Y}a\leq 1,\;\|a{\|}_{1}\leq C_{a},\;b^{⊤}\Sigma_{Z}b\leq 1,\;\|b{\|}_{1,w}\leq C_{b},\;b\in B_{p}.$$ (1)

Here $C_{a},C_{b}>0$ are some positive tuning parameters that control the global shrinkage level. The weight $w_{j}$ allows different penalization strengths according to the data or prior structure information. We will elaborate it in Section 2.3.

It has been shown that in high dimensions, treating the covariance matrix as diagonal can yield good results. Following the same strategy adopted by many of the existing high-dimensional CCA algorithms (e.g., Witten et al., 2009), we substitute in the identity matrix for $\Sigma_{Y}$ and $\Sigma_{Z}$ in the CCA formulation (Equation 1). Moreover, we write the (weighted) $l_{1}$ constraints on a and b in the Lagrangian form. Given a set of n samples ${\left\{X_{i},Y_{i}\right\}}_{i=1}^{n}$ , let ${\hat{\Sigma }}_{YZ}$ be the sample cross-covariance between Y and Z . We formulate the feasible CCA problem as

$$\underset{a\in R^{q},b\in R^{p}}{\min}-a^{⊤}{\hat{\Sigma }}_{YZ}b+\lambda_{a}\|a{\|}_{1}+\lambda_{b}\|b{\|}_{1,w}\;\text{s.t.}\;\|a{\|}_{2}\leq 1,\;\|b{\|}_{2}\leq 1,\;b\in B_{p},$$

which can be solved by iteratively optimizing the objective function with respect to one parameter while fixing the other parameter. Specifically, we have the following two updating steps.

1. **Update** b : Fix $a^{\left(t\right)}$ and update b through

$$b^{\left(t\right)}\leftarrow \underset{b\in R^{p}}{\text{argmin}}-{\left(a^{\left(t\right)}\right)}^{⊤}{\hat{\Sigma }}_{YZ}b+\lambda_{b}\|b{\|}_{1,w}\text{ s.t. }\|b{\|}_{2}\leq 1,\;b\in B_{p}.$$ (2)

2. **Update** a : Fix $b^{\left(t\right)}$ and update a through

$$a^{\left(t+1\right)}\leftarrow \underset{a\in R^{q}}{\text{argmin}}-a^{⊤}{\hat{\Sigma }}_{YZ}b^{\left(t\right)}+\lambda_{a}\|a{\|}_{1}\text{ s.t. }\|a{\|}_{2}\leq 1.$$ (3)

### 2.2 Algorithm

In this section, we discuss the updates in Equations 2, 3. Define the operator

$$g\left(h,\lambda ,w\right)=\underset{b\in B_{p}}{\text{argmin}}\frac{1}{2}\|h-b{\|}_{2}^{2}+\lambda \|b{\|}_{1,w}.$$

By exploring the Karush-Kuhn-Tuchker conditions, we obtain the following result.

Proposition 2.1. *Set* $\overset{˘}{b}={\arg\min}_{b\in B_{p}}-{\left(a^{\left(t\right)}\right)}^{⊤}{\hat{\Sigma }}_{YZ}b+\lambda_{b}\|b{\|}_{1,w}$ *. The solution to* (2) *is given by*

$$b^{\left(t\right)}=\left\{\begin{array}{cc} \overset{˘}{b},\; & \text{if }\|\overset{˘}{b}{\|}_{2}\leq 1,\\ \frac{g\left({\hat{\Sigma }}_{YZ}^{⊤}a^{\left(t\right)},\lambda_{b},w\right)}{\|g\left({\hat{\Sigma }}_{YZ}^{⊤}a^{\left(t\right)},\lambda_{b},w\right){\|}_{2}},\; & \text{if }\|\overset{˘}{b}{\|}_{2}>1. \end{array}\right.$$

Remark 2.1. We employ the augmented Lagrangian method (ALM) to solve the optimization problem in g(h,λ,w) . Specifically, the ALM involves the following two steps of iterations

$$\begin{array}{ccc} & b^{\left(t+1\right)}\leftarrow \underset{b}{\text{argmin}}\frac{1}{2}\|h-b{\|}_{2}^{2}+\lambda_{b}\|b{\|}_{1,w}+\frac{\mu_{1}}{2}{\left(1^{⊤}b+d^{\left(t\right)}\right)}^{2}, & \\ & d^{\left(t+1\right)}\leftarrow d^{\left(t\right)}+\mu_{2}1^{⊤}b^{\left(t+1\right)}, & \end{array}$$

where $\mu_{1},\mu_{2}>0$ are the step sizes in dual gradient ascent, which are set to be 1 in our numerical studies. The optimization problem in the first step can be solved using coordinate descent in an inner loop by iterating across the following p components

$$b_{j}^{\left(t+1,r+1\right)}=\frac{1}{1+\mu_{1}}S\left(h_{j}-\mu_{1}\left(\underset{i<j}{\sum }b_{i}^{\left(t+1,r+1\right)}+\underset{i>j}{\sum }b_{i}^{\left(t+1,r\right)}+d^{\left(t\right)}\right),\lambda_{b}w_{j}\right),$$

where $S\left(a,\lambda \right)=\text{sign}\left(a\right){\left(|a|-\lambda \right)}_{+}$ denotes the soft-thresholding operator and $h_{j}$ is the j th component of h .

Using similar arguments, we can show that the solution to (3) is given by

$$a^{\left(t+1\right)}=\left\{\begin{array}{cc} \overset{˘}{a},\; & \text{if }\|\overset{˘}{a}{\|}_{2}\leq 1,\\ \frac{S\left({\hat{\Sigma }}_{YZ}b^{\left(t\right)},\lambda_{a}\right)}{{\left\|S\left({\hat{\Sigma }}_{YZ}b^{\left(t\right)},\lambda_{a}\right)\right\|}_{2}},\; & \text{if }\|\overset{˘}{a}{\|}_{2}>1, \end{array}\right.$$

where $\overset{˘}{a}={argmin}_{a\in R^{q}}-a^{⊤}{\hat{\Sigma }}_{YZ}b^{\left(t\right)}+\lambda_{a}\|a{\|}_{1}$ and $S\left(a,\lambda \right)={\left(S\left(a_{1},\lambda \right),\ldots ,S\left(a_{p},\lambda \right)\right)}^{⊤}$ . Set $v={\left(v_{1},\ldots ,v_{q}\right)}^{⊤}={\hat{\Sigma }}_{YZ}b^{\left(t\right)}$ . The objective function in the definition of $\overset{˘}{a}$ becomes $\sum_{j=1}^{q}\left(-a_{j}v_{j}+\lambda_{a}|a_{j}|\right)$ . We see that $a_{j}^{\left(t+1\right)}=0$ if $\lambda_{a}\geq v_{j}$ , and $|a_{j}^{\left(t+1\right)}|=\infty$ if $\lambda_{a}<v_{j}$ . Therefore, we have

$$a^{\left(t+1\right)}=\left\{\begin{array}{cc} 0,\; & \text{if }\lambda_{a}\geq \|v{\|}_{\infty },\\ \frac{S\left({\hat{\Sigma }}_{YZ}b^{\left(t\right)},\lambda_{a}\right)}{{\left\|S\left({\hat{\Sigma }}_{YZ}b^{\left(t\right)},\lambda_{a}\right)\right\|}_{2}},\; & \text{if }\lambda_{a}<\|v{\|}_{\infty }. \end{array}\right.$$

As our goal is to find two directions a and b to maximize $\text{Cov}\left(a^{⊤}Y,b^{⊤}Z\right)$ , we only consider the updates when the $l_{2}$ constraints are binding, which leads to Algorithm 1 below.

Algorithm 1. Compositional sCCA: compositional data *versus* non-compositional data.

1. Initialize $a^{\left(0\right)}$ as the first left singular vector with unit $l_{2}$ norm from the singular value decomposition of ${\hat{\Sigma }}_{YZ}$ .
2. **Update** b : Fix $a^{\left(t\right)}$ and update b through
$$b^{\left(t\right)}\leftarrow \frac{g\left({\hat{\Sigma }}_{YZ}^{⊤}a^{\left(t\right)},\lambda_{b},w\right)}{{\left\|g\left({\hat{\Sigma }}_{YZ}^{⊤}a^{\left(t\right)},\lambda_{b},w\right)\right\|}_{2}},$$ (4)
  where $g\left({\hat{\Sigma }}_{YZ}^{⊤}a^{\left(t\right)},\lambda_{b},w\right)$ can be obtained through the iterations described in Remark 2.1.
3. **Update** a : Fix $b^{\left(t\right)}$ and update a through
$$a^{\left(t+1\right)}\leftarrow \frac{S\left({\hat{\Sigma }}_{YZ}b^{\left(t\right)},\lambda_{a}\right)}{{\left\|S\left({\hat{\Sigma }}_{YZ}b^{\left(t\right)},\lambda_{a}\right)\right\|}_{2}}.$$ (5)
4. Iterate Steps 2 and 3 until convergence.

### 2.3 Selecting tuning parameters

To select the regularization parameters $\lambda_{a}$ and $\lambda_{b}$ , we consider a two-stage K -fold cross-validation (CV) method as motivated by Chen et al. (2013). We partition all the samples into M folds, and denote $Y_{k}=Y_{I_{k}}$ and $Z_{k}=Z_{I_{k}}$ , where $I_{k}$ are the indexes of samples in the k th fold for k=1,…,K . The K -fold cross-validation criterion is

$$\text{CV}\left(\lambda_{a},\lambda_{b}\right)=\frac{1}{K}\overset{K}{\underset{k=1}{\sum }}\text{Corr}\left({\hat{a}}_{-k}{\left(\lambda_{a},\lambda_{b}\right)}^{⊤}Y_{k},{\hat{b}}_{-k}{\left(\lambda_{a},\lambda_{b}\right)}^{⊤}Z_{k}\right)$$ (6)

where ${\hat{a}}_{-k}\left(\lambda_{a},\lambda_{b}\right)$ and ${\hat{b}}_{-k}\left(\lambda_{a},\lambda_{b}\right)$ are the solutions to the compositional sCCA problem based on the samples $\left(\cup_{k=1}^{K}I_{k}\right)\backslash I_{k}$ with the tuning parameters $\left(\lambda_{a},\lambda_{b}\right)$ . The parameter selection based on the CV criterion can be influenced by shrinkage problems arising from the sparsity penalty. To avoid this bias, we adopt the coefficients estimated from a two-stage approach when evaluating the CV criterion. In the first stage, we implement Algorithm 1 with the given tuning parameter pair $\left(\lambda_{a},\lambda_{b}\right)$ and exclude variables with zero coefficients. In the second stage, we recalculate the coefficients by applying Algorithm 1 with a tuning parameter pair (0,0). The optimal tuning parameter pair is chosen as the one that maximizes the CV value with these recalculated coefficients. Our approach accounts for the compositional structure in the second stage by restricting the coefficients of compositional data to $B_{p}$ . As will be shown below, the K -fold cross-validation performs reasonably well with K=5 in our numerical studies.

### 2.4 Compositional data *versus* compositional data

We briefly describe an extension to the case, where both X and Y are compositional. For example, we want to associate the composition of the bacterial taxa with that of the fungi taxa. Let $Y={\left(Y_{1},\ldots ,Y_{q}\right)}^{⊤}$ be the relative abundances of another set of compositional features. In this case, we let $U={\left(\log\left(Y_{1}\right),\ldots ,\log\left(Y_{q}\right)\right)}^{⊤}$ and define ${\hat{\Sigma }}_{UZ}$ as the sample covariance between U and Z . Following the derivation in Section 2.1, we formulate the two-sided compositional sCCA problem as

$$\underset{a\in R^{q},b\in R^{p}}{\min}-a^{⊤}{\hat{\Sigma }}_{UZ}b+\lambda_{a}\|a{\|}_{1,w_{1}}+\lambda_{b}\|b{\|}_{1,w_{2}}\;\text{s.t.}\;\|a{\|}_{2}\leq 1,\;\|b{\|}_{2}\leq 1,\;a\in B_{q},b\in B_{p},$$

where $w_{j}=\left(w_{1j},\ldots ,w_{pj}\right)$ for j=1,2 are non-negative weights. This problem can be solved by Algorithm 2 below. We use the two-stage CV criterion to select the tuning parameters in the same way as described in Section 2.3.

Algorithm 2. Compositional sCCA: compositional data *versus* compositional data.

1. Initialize $a^{\left(0\right)}$ as the first left singular vector with unit $l_{2}$ norm from the singular value decomposition of ${\hat{\Sigma }}_{UZ}$ .
2. **Update** b : Fix $a^{\left(t\right)}$ and update b through
$$b^{\left(t\right)}\leftarrow \frac{g\left({\hat{\Sigma }}_{UZ}^{⊤}a^{\left(t\right)},\lambda_{b},w_{2}\right)}{\|g\left({\hat{\Sigma }}_{UZ}^{⊤}a^{\left(t\right)},\lambda_{b},w_{2}\right){\|}_{2}}.$$ (7)
3. **Update** a : Fix $b^{\left(t\right)}$ and update a through
$$a^{\left(t+1\right)}\leftarrow \frac{g\left({\hat{\Sigma }}_{UZ}b^{\left(t\right)},\lambda_{a},w_{1}\right)}{{\left\|g\left({\hat{\Sigma }}_{UZ}b^{\left(t\right)},\lambda_{a},w_{1}\right)\right\|}_{2}}.$$ (8)
4. Iterate Steps 2 and 3 until convergence.

## 3 Structure-adaptive compositional sCCA

In this section, following the strategy proposed in Pramanik and Zhang (2020), we aim to incorporate the prior structure information robustly in the compositional sCCA procedure. The prior structure information could be the grouping structure or the phylogenetic tree structure among the taxa. The idea is to define a set of constraints that encode the prior structure information and use the constraints together with the data to estimate the weights w in an iterative fashion. It is worth mentioning that our constraint is “soft” as compared to the “hard” constraints used by traditional approaches such as the group Lasso or fused Lasso. As a result, our method provides significant improvement when an external structure is informative while maintaining robustness against a misspecified structure.

### 3.1 Structure-adaptive weights

Based on the setups described in Section 2.1, our procedure is to translate the auxiliary information into different penalization strengths through the weights w . Our framework is general enough to incorporate different types of external structures. For instance, co-expressed genes can be classified into the same group to reflect their biological relationships. In our study, we focus on leveraging the taxonomic grouping structure among taxa. Taxonomically related taxa, such as multiple species within the same genus, tend to share biological traits. Consequently, we anticipate that these taxa will exhibit similar relationships with omics features, leading to the expectation of comparable CCA coefficients. We translate the grouping structure information into different restrictions on the weights. Specifically, we divide the taxa into different groups according to their taxonomy such as phylum, family, and genus. We next consider the set of weights:

$$M_{\text{Group}}=\{w\in {\left[0,C_{U}\right]}^{p}:w_{i}=w_{j}\text{ if }i,j\in S_{d}\text{ for }i,j\in \left\{1,2,\ldots ,p\right\}\text{ and }d\in \left\{1,2,\ldots ,D\right\}\},$$

where D represents the number of groups and $C_{U}$ denotes an upper bound on the weights.

### 3.2 Structure-adaptive compositional sCCA

Following Pramanik and Zhang (2020), we impose a penalty term on the weights and propose an algorithm to jointly estimate weights and parameters. Specifically, we define

$$h\left(w_{j};\gamma \right)=\left\{\begin{array}{cc} \exp\left\{w_{j}^{1-\frac{1}{\gamma }}/\left(1-\frac{1}{\gamma }\right)\right\},\; & \text{if }0<\gamma <1,\\ w_{j},\; & \text{if }\gamma =1. \end{array}\right.$$

We estimate (a,b) and w jointly by solving the following problem

$$\begin{array}{ccc} & \underset{a\in R^{q},b\in R^{p},w\in M}{\min}-a^{⊤}{\hat{\Sigma }}_{YZ}b+\lambda_{a}\|a{\|}_{1}+\lambda_{b}\overset{p}{\underset{j=1}{\sum }}\left\{w_{j}|b_{j}|-\log h\left(w_{j},\gamma \right)\right\}\; & \\ & \text{s.t.}\;\|a{\|}_{2}\leq 1,\;\|b{\|}_{2}\leq 1,\;b\in B_{p}. & \end{array}$$

The design of the function h is to reduce our method to the classic (iterative) adaptive Lasso when there is no external information. The readers are referred to Pramanik and Zhang (2020) for more discussions on the motivation.

Next we introduce the algorithm to solve the above problem. We focus on the update for w as the updates for a and b remain the same as in Section 2.2. In particular, we update w through

$$w^{\left(t+1\right)}\leftarrow \underset{w\in M}{\text{argmin}}\overset{p}{\underset{j=1}{\sum }}\left\{w_{j}|b_{j}^{\left(t\right)}|-\log h\left(w_{j},\gamma \right)\right\}.$$

When $M=M_{\text{Group}}$ , it is straightforward to verify that

$$w_{j}^{\left(t+1\right)}=\left\{\begin{array}{cc} C_{U}\; & \text{ if }b_{j}^{\left(t\right)}=0\text{ for all }j\in S_{d},\\ {\left(\frac{1}{\left|S_{d}{|}^{-1}\sum_{j\in S_{d}}|b_{j}^{\left(t\right)}\right|}\right)}^{\gamma }\; & \text{ otherwise}. \end{array}\right.$$

If we do not have any prior structural information on b that we can take advantage of, we take M to be ${\left[0,C_{U}\right]}^{p}$ . In this case, we have

$$w_{j}^{\left(t+1\right)}=\left\{\begin{array}{cc} C_{U}\; & \text{ if }b_{j}^{\left(t\right)}=0,\\ |b_{j}^{\left(t\right)}{|}^{-\gamma }\; & \text{ otherwise}. \end{array}\right.$$

Algorithm 3 summarizes the implementation details of the structure adaptive Compositional sCCA. The selection of tuning parameter pair $\left(\lambda_{a},\lambda_{b},\gamma \right)$ follows a similar approach as described in Section 2.3, with the difference of using $\left(\lambda_{a},\lambda_{b},\gamma \right)$ in the first stage and (0,0,γ) in the second stage.

Algorithm 3. Structure-Adaptive Compositional sCCA: compositional data *versus* non-compositional data.

1. Initialize $a^{\left(0\right)}$ as the first left singular vector with unit $l_{2}$ norm from the singular value decomposition of ${\hat{\Sigma }}_{YZ}$ .
2. **Update** b : Fix $a^{\left(t\right)}$ and update b through
$$b^{\left(t\right)}\leftarrow \frac{g\left({\hat{\Sigma }}_{YZ}^{⊤}a^{\left(t\right)},\lambda_{b},w^{\left(t\right)}\right)}{\|g\left({\hat{\Sigma }}_{YZ}^{⊤}a^{\left(t\right)},\lambda_{b},w^{\left(t\right)}\right){\|}_{2}},$$ (9)
  where $g\left({\hat{\Sigma }}_{YZ}^{⊤}a^{\left(t\right)},\lambda_{b},w^{\left(t\right)}\right)$ can be obtained through the iterations described in Remark 2.1.
3. **Update** w : Fix $b^{\left(t\right)}$ and update w through
$$w^{\left(t+1\right)}\leftarrow \underset{w\in M}{\text{argmin}}\overset{p}{\underset{j=1}{\sum }}\left\{w_{j}|b_{j}^{\left(t\right)}|-\log h\left(w_{j},\gamma \right)\right\}.$$
4. **Update** a : Fix $b^{\left(t\right)}$ and update a through
$$a^{\left(t+1\right)}\leftarrow \frac{S\left({\hat{\Sigma }}_{YZ}b^{\left(t\right)},\lambda_{a}\right)}{{\left\|S\left({\hat{\Sigma }}_{YZ}b^{\left(t\right)},\lambda_{a}\right)\right\|}_{2}}.$$ (10)
5. Iterate Steps 2-4 until convergence.

## 4 Simulation studies

In this section, we evaluate the finite sample performance of the proposed methods through numerical simulations.

### 4.1 Compositional data *versus* non-compositional data

We first consider the CCA problem between compositional data (i.e., microbiome data) and non-compositional data (e.g., metabolomics data) following a similar setting considered in Chen et al. (2013). To capture the dependence between the two sets of high-dimensional data, we use a latent variable model to generate the compositional variables $\left\{X_{i}\right\}$ (log scale) and non-compositional variables $\left\{Y_{i}\right\}$ (original scale), where the dependence between these two sets of variables is governed by a latent variable ν . Specifically, we assume that

$$\log\left(X_{i}\right)=\nu_{i}\omega_{X}+\varepsilon_{X,i},\;Y_{i}=\nu_{i}\omega_{Y}+\varepsilon_{Y,i},$$

where $\nu_{i}∼N\left(0,\sigma_{\nu }^{2}\right)$ and $\varepsilon_{X,i},\varepsilon_{Y,i}$ follow $N\left(0_{p},\sigma_{\varepsilon }^{2}I_{p\times p}\right)$ and $N\left(0_{q},\sigma_{\varepsilon }^{2}I_{q\times q}\right)$ , respectively. The coefficients $\omega_{X}\in R^{p}$ and $\omega_{Y}\in R^{q}$ control the relative contributions of individual variables to the overall association. The ratio $\sigma_{\nu }/\sigma_{\varepsilon }$ determines the overall association strength between log(X) and Y , with a larger value indicating stronger association. For the dimensions, we set (p,q)=(100,100),(200,200) . We consider two setups for $\omega_{X}$ .

S1 $\omega_{X}=\frac{0.85}{10}\times {\left(1,1,1,1,1,1,1,1,1-9,0_{p-10}\right)}^{⊤}$ ;
S2 $\omega_{X}=\frac{0.85}{6}\times {\left(1,1,1,0,0,1,1,-5,0,0,0_{p-10}\right)}^{⊤}$ ;

where $1_{p}^{⊤}\omega_{X}=0$ for both setups. The constraints imply that the association between X and Y is mediated through the log ratios for X . We focus on the group structure (i.e., $M=M_{\text{group}}$ ) and assume that the p taxa form 20 groups, with the group size equal to 5 for p=100 and equal to 10 for p=200 . For example, in Setup S2 with p=100 , the grouping is defined as

$$\omega_{X}=\frac{0.85}{6}\times {\left(\underset{\text{Group }1}{\underbrace{1,1,1,0,0}},\underset{\text{Group }2}{\underbrace{1,1,-5,0,0}},\underset{\text{Groups }3-20}{\underbrace{0_{p-10}}}\right)}^{⊤}$$

The first two groups contain both zero and nonzero entries reflecting the fact that the external structure information is imperfect and noisy. We set $\omega_{Y}=0.85\times {\left(0.08,0.084,0.089,\ldots ,0.12,0_{q-10}^{⊤}\right)}^{⊤}$ . Next, we fix $\sigma_{\varepsilon }=1$ and vary $\sigma_{\nu }$ within {1,2,…,8} to control the strength of the canonical correlation. We report the true positive rate (TPR), false positive rate (FPR), Matthew’s correlation coefficient (MCC), and Precision to measure the performance of different methods. Here,

$$\text{TPR}=\frac{\text{TP}}{\text{TP}+\text{FN}},\;\text{FPR}=\frac{\text{FP}}{\text{FP}+\text{TN}},\;\text{MCC}=\frac{\text{TP}\times \text{TN}-\text{FP}\times \text{FN}}{\sqrt{\left(\text{TP}+\text{FP}\right)\left(\text{TP}+\text{FN}\right)\left(\text{TN}+\text{FP}\right)\left(\text{TN}+\text{FN}\right)}},\;\text{Precision}=\frac{\text{TP}}{\text{FP}+\text{TP}},$$

where TP, FP, TN, and FN represent the true positives, false positives, true negatives, and false negatives, respectively. The TPR, FPR, MCC, and Precision are computed by averaging over 100 simulation replicates. Denote the estimated canonical coefficients by $\hat{a}$ and $\hat{b}$ . Their estimation targets are $\omega_{X}/\|\omega_{X}{\|}_{2}$ and $\omega_{Y}/\|\omega_{Y}{\|}_{2}$ , respectively, where this normalization is to ensure comparability. The estimation accuracy is evaluated using the root mean square error (RMSE).

We compare the performance of the following four methods.

1. sCCA: sCCA without considering the compositional effect;
2. C-sCCA: compositional sCCA;
3. AC-sCCA: adaptive compositional sCCA, i.e., $M={\left[0,C_{U}\right]}^{p}$ .
4. SAC-sCCA: structure adaptive compositional sCCA, i.e., $M=M_{\text{Group}}$ .

For AC-sCCA and SAC-sCCA, we also apply adaptive weights on a with $M={\left[0,C_{U}\right]}^{p}$ in implementation. The value of $C_{U}$ is set to $10^{5}$ .

**FIGURE 1:**
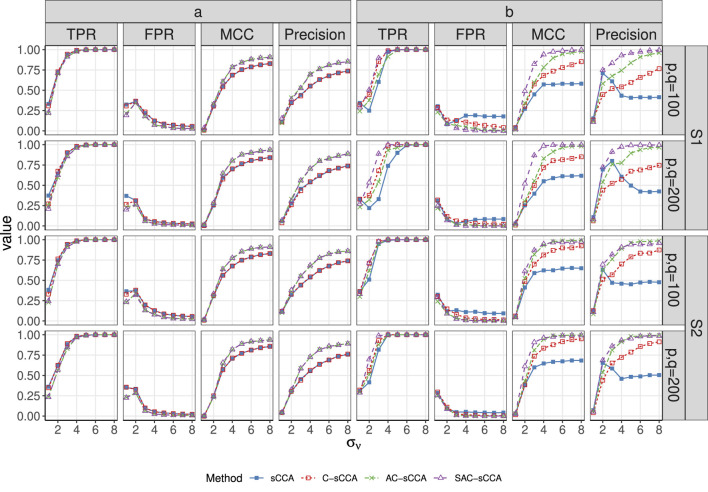
TPR, FPR, MCC, and Precision of sCCA for the association between compositional data and non-compositional data across association strength. Here, the range of $\sigma_{\nu }$ is {1,2,…,8} . Line with solid blue squares: sCCA; line with open red squares: C-sCCA; line with green crosses: AC-sCCA; line with open purple triangles: SAC-sCCA.

**FIGURE 2:**
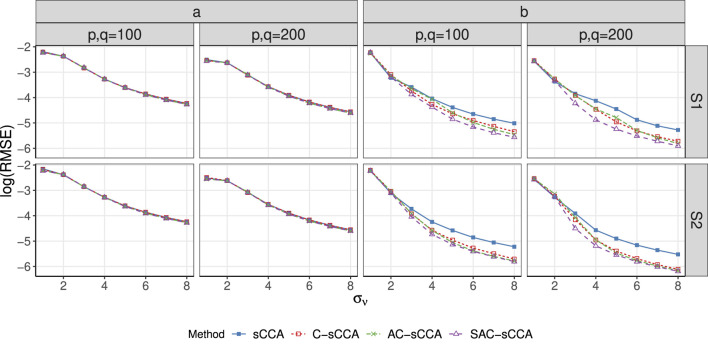
RMSE of sCCA for the association between compositional data and non-compositional data across association strength. Here, the range of $\sigma_{\nu }$ is {1,2,…,8} . Line with solid blue squares: sCCA; line with open red squares: C-sCCA; line with green crosses: AC-sCCA; line with open purple triangles: SAC-sCCA.

Table 1 summarizes the results for the above four methods when fixing $\sigma_{\nu }=4$ . For both Setups S1 and S2, C-sCCA outperforms sCCA in terms of all four measures, especially in reducing the false positive rates and increasing the precision in identifying relevant compositional components, demonstrating the advantage of taking into account the compositional constraint. Compared to the first two methods, AC-sCCA and SAC-sCCA further reduce the FPR and thus lead to higher MCC in estimating a and b . For identifying b in Setup S1, SAC-sCCA outperforms the other three methods by exhibiting higher TPR, nearly zero FPR, and thus higher MCC because of incorporating grouping information. Figure 1 is in general consistent with these findings. As association strength increases, the TPR, MCC, and Precision of C-sCCA, AC-sCCA, and SAC-sCCA increase, whereas their FPRs show a declining trend. When $\sigma_{\nu }\geq 3$ , the precision and FPR in estimating b of sCCA becomes worse as association strength increases, which means sCCA identifies more true variables at the cost of including more false variables. Figure 2 presents the RMSE in estimating the canonical coefficients, which decreases as the association strength $\sigma_{\nu }$ increases. By accounting for the compositional effect, the C-sCCA, AC-sCCA, and SAC-sCCA outperform sCCA, with SAC-sCCA providing the most accurate estimation. This demonstrates that methods accounting for the compositional nature yield more accurate estimations than those that do not, and considering structural information can further enhance performance.

**TABLE 1:** Performance of sCCA for the association between compositional data and non-compositional data $\left(\sigma_{\nu }=4\right)$ . Numbers in the parentheses represent the corresponding standard deviations.

Setup	p=q	Method	$\hat{a}$				$\hat{b}$			
			TPR	FPR	MCC	Precision	TPR	FPR	MCC	Precision
S1	100	sCCA	0.99 (0.03)	0.13 (0.13)	0.68 (0.16)	0.55 (0.19)	0.97 (0.11)	0.19 (0.14)	0.57 (0.15)	0.43 (0.18)
		C-sCCA	0.99 (0.03)	0.12 (0.12)	0.69 (0.16)	0.55 (0.19)	0.99 (0.09)	0.11 (0.06)	0.68 (0.11)	0.54 (0.14)
		AC-sCCA	0.98 (0.06)	0.07 (0.12)	0.79 (0.17)	0.70 (0.21)	0.92 (0.18)	0.05 (0.06)	0.78 (0.16)	0.74 (0.19)
		SAC-sCCA	0.98 (0.05)	0.07 (0.11)	0.78 (0.16)	0.69 (0.21)	0.98 (0.13)	0.01 (0.03)	0.94 (0.13)	0.93 (0.14)
	200	sCCA	0.98 (0.05)	0.06 (0.04)	0.70 (0.12)	0.54 (0.16)	0.74 (0.38)	0.06 (0.05)	0.55 (0.17)	0.61 (0.28)
		C-sCCA	0.98 (0.04)	0.05 (0.04)	0.70 (0.12)	0.54 (0.16)	0.99 (0.04)	0.05 (0.04)	0.72 (0.16)	0.57 (0.22)
		AC-sCCA	0.96 (0.06)	0.03 (0.03)	0.80 (0.12)	0.70 (0.18)	0.93 (0.13)	0.02 (0.02)	0.83 (0.12)	0.78 (0.16)
		SAC-sCCA	0.96 (0.06)	0.03 (0.03)	0.80 (0.11)	0.71 (0.17)	1.00 (0.00)	0.00 (0.00)	0.99 (0.04)	0.97 (0.07)
S2	100	sCCA	0.99 (0.03)	0.13 (0.12)	0.67 (0.16)	0.54 (0.18)	0.99 (0.08)	0.11 (0.09)	0.62 (0.17)	0.46 (0.22)
		C-sCCA	0.99 (0.03)	0.13 (0.12)	0.68 (0.16)	0.54 (0.19)	1.00 (0.00)	0.04 (0.05)	0.81 (0.14)	0.70 (0.21)
		AC-sCCA	0.98 (0.05)	0.08 (0.12)	0.77 (0.17)	0.67 (0.21)	1.00 (0.02)	0.01 (0.03)	0.93 (0.12)	0.89 (0.18)
		SAC-sCCA	0.98 (0.05)	0.08 (0.12)	0.78 (0.16)	0.68 (0.21)	1.00 (0.00)	0.01 (0.01)	0.95 (0.07)	0.91 (0.12)
	200	sCCA	0.98 (0.04)	0.06 (0.04)	0.71 (0.14)	0.55 (0.19)	1.00 (0.00)	0.05 (0.04)	0.65 (0.15)	0.46 (0.19)
		C-sCCA	0.98 (0.04)	0.05 (0.04)	0.71 (0.13)	0.56 (0.18)	1.00 (0.00)	0.02 (0.02)	0.84 (0.13)	0.72 (0.19)
		AC-sCCA	0.97 (0.06)	0.03 (0.03)	0.82 (0.13)	0.72 (0.20)	0.99 (0.08)	0.00 (0.01)	0.95 (0.09)	0.91 (0.13)
		SAC-sCCA	0.97 (0.06)	0.03 (0.03)	0.82 (0.13)	0.73 (0.20)	1.00 (0.00)	0.00 (0.00)	0.96 (0.06)	0.93 (0.10)

Finally, we examine the scenario where p=100 and q=200 to assess the performance of our method on unbalanced datasets. As presented in Table 2, the four methods exhibit similar performance to that observed in the case where p=q . The results indicate that our method successfully handles unbalanced dimensions.

**TABLE 2:** Performance of sCCA for the association between compositional data and non-compositional data ( $\sigma_{\nu }=4$ , p=100 , q=200 ). Numbers in the parentheses represent the corresponding standard deviations.

Setup	Method	$\hat{a}$				$\hat{b}$			
		TPR	FPR	MCC	Precision	TPR	FPR	MCC	Precision
S1	sCCA	0.98 (0.04)	0.05 (0.05)	0.74 (0.15)	0.61 (0.22)	0.92 (0.21)	0.16 (0.11)	0.58 (0.15)	0.48 (0.22)
	C-sCCA	0.99 (0.04)	0.05 (0.05)	0.75 (0.15)	0.61 (0.22)	0.98 (0.09)	0.10 (0.05)	0.70 (0.10)	0.56 (0.13)
	AC-sCCA	0.97 (0.06)	0.02 (0.03)	0.85 (0.12)	0.77 (0.19)	0.93 (0.16)	0.04 (0.04)	0.80 (0.15)	0.75 (0.18)
	SAC-sCCA	0.97 (0.06)	0.02 (0.03)	0.85 (0.12)	0.78 (0.19)	0.98 (0.13)	0.01 (0.02)	0.95 (0.12)	0.94 (0.12)
S2	sCCA	0.98 (0.04)	0.05 (0.04)	0.73 (0.13)	0.58 (0.18)	0.99 (0.08)	0.11 (0.08)	0.63 (0.17)	0.47 (0.22)
	C-sCCA	0.98 (0.04)	0.05 (0.04)	0.73 (0.13)	0.58 (0.18)	1.00 (0.00)	0.04 (0.04)	0.82 (0.15)	0.71 (0.23)
	AC-sCCA	0.97 (0.06)	0.02 (0.02)	0.84 (0.11)	0.75 (0.18)	1.00 (0.00)	0.01 (0.02)	0.95 (0.09)	0.92 (0.14)
	SAC-sCCA	0.97 (0.06)	0.02 (0.02)	0.84 (0.12)	0.75 (0.19)	1.00 (0.00)	0.01 (0.01)	0.96 (0.07)	0.93 (0.11)

### 4.2 Compositional data *versus* compositional data

In this section, we study the performance of the proposed compositional sCCA for the association between two compositional datasets, for example, bacterial taxa abundance vs fungi taxa abundance. We modify the setting in Section 4.1 by considering the following models

$$\log\left(X_{i}\right)=\nu_{i}\omega_{X}+\varepsilon_{X,i},\;\log\left(Y_{i}\right)=\nu_{i}\omega_{Y}+\varepsilon_{Y,i},$$

where $\nu_{i}∼N\left(0,\sigma_{\nu }^{2}\right)$ and $\varepsilon_{X,i},\varepsilon_{Y,i}$ follow $N\left(0_{p},\sigma_{\varepsilon }^{2}I_{p\times p}\right)$ and $N\left(0_{q},\sigma_{\varepsilon }^{2}I_{q\times q}\right)$ , respectively. We again consider two setups for $\omega_{X}$ and $\omega_{Y}$ .

S3 $\omega_{X}=\frac{0.85}{10}\times {\left(1_{9}^{⊤},-9,0_{p-10}\right)}^{⊤}\text{ and }\omega_{Y}=0.85\times {\left(0.08,0.085,0.09,\ldots ,0.12,-0.9,0_{q-10}^{⊤}\right)}^{⊤};$

**FIGURE 3:**
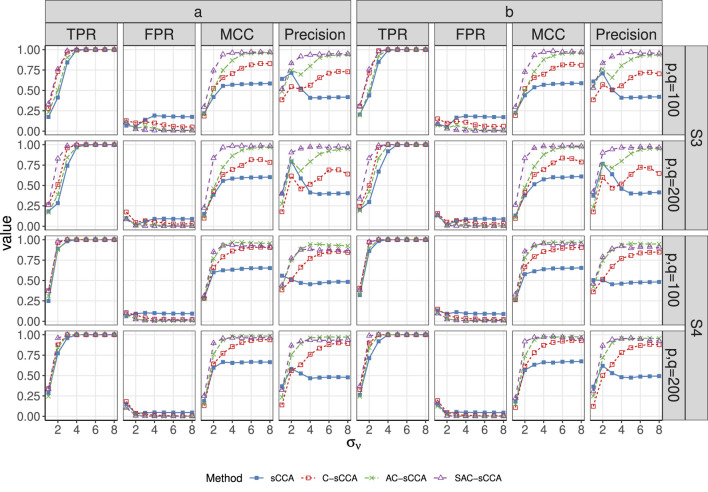
TPR, FPR, MCC, and Precision of sCCA for the association between two compositional datasets across signal strength. Here, the range of $\sigma_{\nu }$ is {1,2,…,8} . Line with solid blue squares: sCCA; line with open red squares: C-sCCA; line with green crosses: AC-sCCA; line with open purple triangles: SAC-sCCA.

**FIGURE 4:**
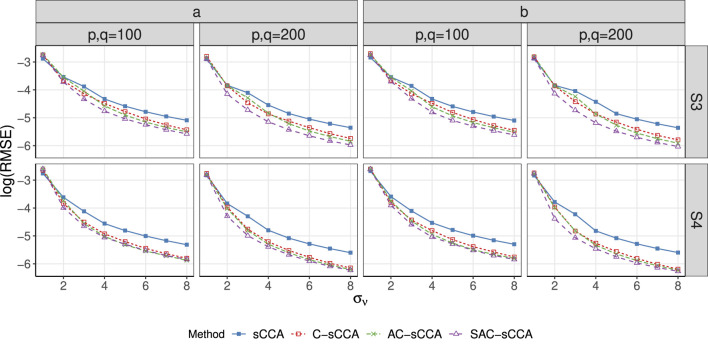
RMSE of sCCA for the association between two compositional datasets across association strength. Here, the range of $\sigma_{\nu }$ is {1,2,…,8} . Line with solid blue squares: sCCA; line with open red squares: C-sCCA; line with green crosses: AC-sCCA; line with open purple triangles: SAC-sCCA.

S4 $\omega_{X}=\frac{0.85}{6}\times {\left(1,1,1,0,0,1,1,-5,0,0,0_{p-10}\right)}^{⊤}\text{ and }\omega_{Y}=\frac{0.85}{6}\times {\left(1,1,1,0,0,1,1,-5,0,0,0_{q-10}\right)}^{⊤};$

where $1_{p}^{⊤}\omega_{X}=0$ and $1_{q}^{⊤}\omega_{Y}=0$ for both setups. We group $\omega_{X}$ and $\omega_{Y}$ using the same strategy applied to $\omega_{X}$ in Section 4.1. The other setups are the same as those in Section 4.1. Table 3 summarizes the empirical results. For Setups S3 and S4, C-sCCA often results in higher TPR, higher Precision, lower FPR and therefore higher MCC in estimating both a and b compared to sCCA, emphasizing the necessity of including compositional constraints again. For Setup S3, SAC-sCCA significantly outperforms the other methods in terms of higher TPR and MCC. Our structure-adaptive approach is robust against a misspecified structure (Setup S4) as the performance of SAC-sCCA is comparable to that of AC-sCCA. Figures 3, 4 show patterns similar to Figures 1, 2.

**TABLE 3:** Performance of sCCA for the association between two compositional datasets $\left(\sigma_{\nu }=4\right)$ . Numbers in the parentheses represent the corresponding standard deviations.

Setup	p=q	Method	$\hat{a}$				$\hat{b}$			
			TPR	FPR	MCC	Precision	TPR	FPR	MCC	Precision
S3	100	sCCA	1.00 (0.02)	0.19 (0.12)	0.57 (0.13)	0.41 (0.13)	0.99 (0.09)	0.18 (0.09)	0.57 (0.11)	0.41 (0.13)
		C-sCCA	0.99 (0.02)	0.10 (0.06)	0.71 (0.11)	0.56 (0.15)	1.00 (0.01)	0.11 (0.08)	0.70 (0.13)	0.56 (0.17)
		AC-sCCA	0.99 (0.03)	0.03 (0.03)	0.87 (0.10)	0.80 (0.15)	1.00 (0.01)	0.03 (0.03)	0.88 (0.10)	0.80 (0.15)
		SAC-sCCA	1.00 (0.01)	0.01 (0.02)	0.96 (0.07)	0.94 (0.11)	1.00 (0.00)	0.01 (0.03)	0.97 (0.08)	0.96 (0.12)
	200	sCCA	0.97 (0.14)	0.09 (0.05)	0.59 (0.11)	0.41 (0.17)	0.92 (0.25)	0.08 (0.06)	0.58 (0.13)	0.46 (0.21)
		C-sCCA	0.99 (0.03)	0.05 (0.02)	0.69 (0.07)	0.52 (0.09)	1.00 (0.00)	0.06 (0.03)	0.69 (0.10)	0.52 (0.13)
		AC-sCCA	0.97 (0.05)	0.02 (0.02)	0.86 (0.10)	0.79 (0.15)	0.99 (0.03)	0.02 (0.01)	0.88 (0.09)	0.80 (0.15)
		SAC-sCCA	1.00 (0.01)	0.00 (0.00)	0.98 (0.04)	0.97 (0.07)	1.00 (0.00)	0.00 (0.01)	0.98 (0.06)	0.97 (0.09)
S4	100	sCCA	1.00 (0.00)	0.10 (0.07)	0.63 (0.14)	0.45 (0.17)	1.00 (0.00)	0.10 (0.07)	0.64 (0.14)	0.46 (0.17)
		C-sCCA	1.00 (0.00)	0.03 (0.03)	0.86 (0.12)	0.77 (0.19)	1.00 (0.00)	0.03 (0.06)	0.85 (0.16)	0.77 (0.23)
		AC-sCCA	1.00 (0.00)	0.00 (0.01)	0.97 (0.06)	0.94 (0.10)	1.00 (0.00)	0.01 (0.01)	0.96 (0.08)	0.94 (0.12)
		SAC-sCCA	1.00 (0.00)	0.01 (0.01)	0.93 (0.08)	0.89 (0.13)	1.00 (0.00)	0.01 (0.01)	0.95 (0.07)	0.92 (0.11)
	200	sCCA	1.00 (0.00)	0.05 (0.04)	0.66 (0.14)	0.47 (0.19)	1.00 (0.00)	0.05 (0.04)	0.66 (0.16)	0.48 (0.21)
		C-sCCA	1.00 (0.00)	0.02 (0.03)	0.86 (0.15)	0.76 (0.22)	1.00 (0.00)	0.01 (0.01)	0.87 (0.12)	0.78 (0.20)
		AC-sCCA	1.00 (0.00)	0.00 (0.00)	0.98 (0.05)	0.96 (0.09)	1.00 (0.00)	0.00 (0.00)	0.97 (0.05)	0.95 (0.09)
		SAC-sCCA	1.00 (0.00)	0.00 (0.01)	0.96 (0.07)	0.94 (0.11)	1.00 (0.00)	0.00 (0.00)	0.97 (0.05)	0.95 (0.10)

## 5 Real application

We applied sCCA, C-sCCA, AC-sCCA and SAC-sCCA to examine the association between gut bacterial composition and gut metabolism in a colorectal adenoma study conducted at the Mayo Clinic. The study utilized both gut microbiome and gut metabolomics data from 241 fecal samples selected from a frozen stool archive. The fecal samples were collected following a standard protocol and metabolomics profiling was conducted by Metabolon, Inc. (Durham, NC, United States) using a UPLCMS/MS platform, as detailed in Kim et al. (2020). Metabolic sub-pathway abundances were calculated by averaging the scaled abundances of metabolites within each sub-pathway, which are grouped into super-pathways. Bacterial DNA extraction and 16S rRNA gene sequencing were described in Hale et al. (2017). Specifically, the sequencing library was prepared at the University of Minnesota Genomics Center, and sequencing was performed using the Illumina MiSeq system at the Mayo Clinic Medical Genome Facility. These sequences were processed through the IM-TORNADO bioinformatics pipeline, clustering them into OTUs based on a 97% identity threshold.

We focused the analysis on the overall association between the bacterial genera and metabolic sub-pathways. We followed Chen et al. (2013) to pre-process data. We excluded genera that were present in less than 1/4 samples and kept 63 relatively common genera, each belonging to a specific phylum. This approach ensures a balance between retaining sufficient taxa for meaningful analysis while filtering out rare genera that could introduce noise. Zeros were replaced with 0.5 in microbiome data to facilitate working on the log scale. Our final dataset is summarized as a metabolic sub-pathway abundance matrix $Y_{\mathrm{241\times 91}}$ and a bacterial genus abundance matrix $X_{\mathrm{241\times 63}}$ . The group information, specified as super-pathway and phylum, respectively, is incorporated into our analysis. We applied logarithmic transformation to both matrices: for metabolic data, to normalize the distribution, and for genus abundance, to account for the compositional structure. Finally, we performed standardization to ensure that all variables have zero mean and unit variance.

**FIGURE 5:**
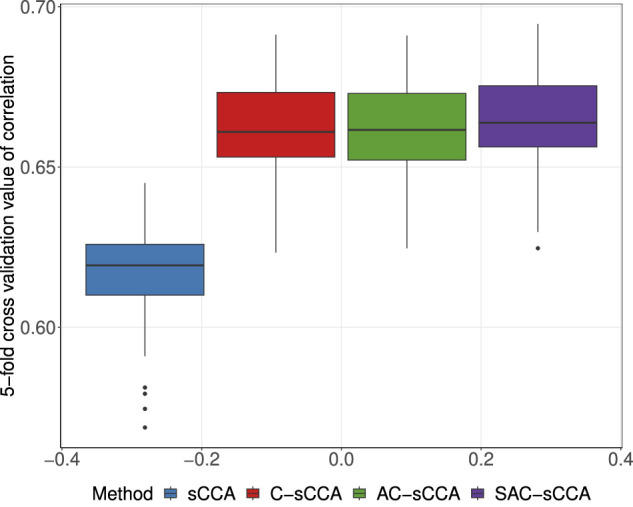
Boxplot of 5-fold cross-validated correlations for sCCA, C-sCCA, AC-sCCA, and SAC-sCCA across 100 replications, with tuning parameters determined by sample partition in each replication.

We performed a two-stage five-fold CV described in Section 2.3 to identify the optimal tuning parameters across a range of models, from the most dense to the most sparse. To mitigate randomness, we conducted 100 replications of sample partitions. We selected the tuning parameter pair for each replication and recorded the corresponding CV values of four methods. As shown in Figure 5, sCCA has the lowest CV correlations, followed by C-sCCA and AC-sCCA, both of which yield comparable CV correlations, while SAC-sCCA achieves slightly higher CV correlations by incorporating grouping information. Therefore, by accounting for the compositional structure, we achieved a stronger association between the two datasets. The final parameters were determined by maximizing the CV values averaged across the 100 replications, with CV values of 0.6076 for sCCA, 0.6578 for C-sCCA, 0.6581 for AC-sCCA, and 0.6584 for SAC-sCCA.

**FIGURE 6:**
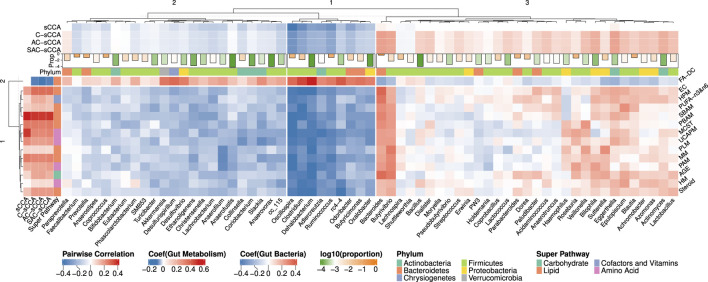
Heatmap of Spearman correlations between the bacterial genera and metabolic sub-pathways selected by either sCCA, C-sCCA, AC-sCCA, or SAC-sCCA. The color indicates the association direction, with red for positive correlations and blue for negative, varying in shade by strength. The bars at the top represent the average relative abundances of these genera on a log 10 scale, with orange indicating higher values and green indicating lower values. Abbreviations: AGE (Advanced Glycation End-product), EC (Endocannabinoid), FA-DC (Fatty Acid, Dicarboxylate), HPM (Hemoglobin and Porphyrin Metabolism), HM (Histidine Metabolism), MCST (Methionine, Cysteine, SAM and Taurine Metabolism), MM (Mevalonate Metabolism), PLM (Phospholipid Metabolism), PAM (Polyamine Metabolism), PUFA-n3&n6 (Polyunsaturated Fatty Acid, n3 and n6), PBAM (Primary Bile Acid Metabolism), SBAM (Secondary Bile Acid Metabolism), Steroid (Steroid), UCAPM (Urea cycle; Arginine and Proline Metabolism).

Figure 6 shows the heatmap of pairwise spearman correlations between metabolic sub-pathways and genera selected by any of the four methods. The signs of the estimated coefficients align with the pairwise correlations. The selected metabolic sub-pathways belong to four super-pathways: *Carbohydrate*, *Lipid*, *Cofactors and Vitamins*, and *Amino Acid*. Hierarchical clustering analysis, using the complete linkage and Euclidean distance, was applied to cluster the bacterial genera. The coefficients estimated by C-sCCA, AC-sCCA, and SAC-sCCA for bacteria within the third cluster were mostly positive while the other two clusters showed an opposite trend. Interestingly, *Fatty Acid, Diacarboxylate (FA-DC)*, identified by C-sCCA, AC-sCCA, and SAC-sCCA but not by sCCA, was overall negatively correlated with bacterial genera in the third cluster, and positively correlated with those in the first/second clusters. Dicarboxylic acids can be produced by various bacteria through different metabolic pathways (Yu et al., 2018). For example, species in the genus *Clostridium*, which showed a strong correlation with FA-DC in our data, can produce succinic acid and other dicarboxylic acids as fermentation products (Koendjbiharie et al., 2018). As a comparison, other detected metabolic sub-pathways exhibited both negative and positive correlations with bacterial genera in the third cluster, and overall negative correlations with those in the first/second clusters.

**FIGURE 7:**
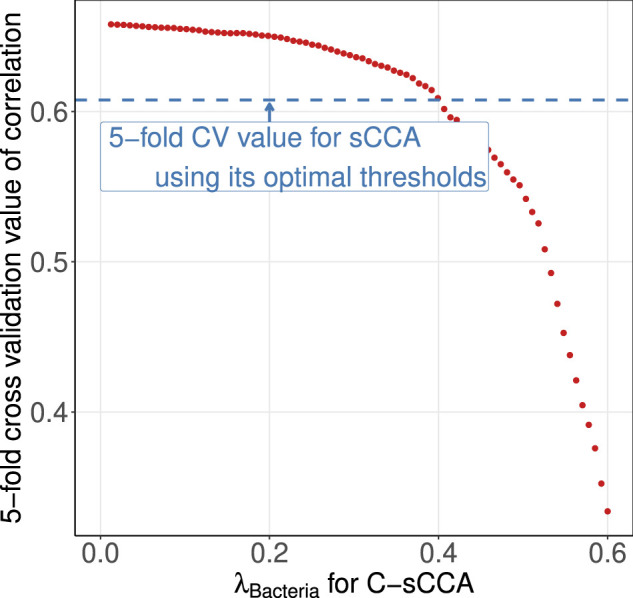
Averaged cross-validated correlations across 100 replications. The blue horizontal line represents the CV value of sCCA with its optimal threshold. The red points denotes the CV values of C-sCCA with varying tuning parameters for gut bacterial genera.

Despite achieving higher cross-validated correlations, the three methods that accounted for compositional structure failed to induce a sparse structure for the bacterial genera. Although this may be the biological truth, as gut metabolic capabilities are contributed by a large number of bacteria collectively (Cox et al., 2022), to gain more insights into the benefits of using the compositional constraint, we reconsidered C-sCCA by fixing the final parameter for metabolic sub-pathways and varying the parameter for bacterial genera to achieve the same cross-validated correlation as sCCA. Figure 7 shows the average cross-validated correlation of C-sCCA for each parameter pair. We selected the tuning parameter of bacterial genera as 0.4 so that the averaged CV value of C-sCCA is almost the same as that of sCCA.

**FIGURE 8:**
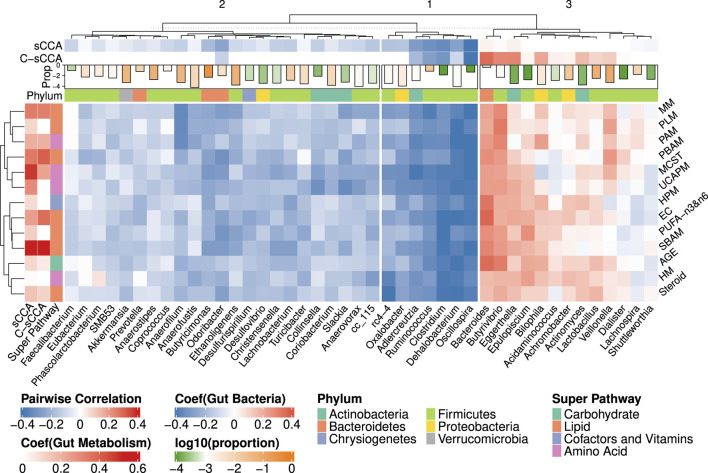
Heatmap of Spearman correlations between the bacterial genera and metabolic sub-pathways selected by either sCCA or C-sCCA. The CV values averaged across 100 replications are approximately 0.61 for both methods with the chosen tuning parameters. Other details are the same as in Figure 6.

Figure 8 presents the heatmap of pairwise Spearman correlations between the metabolic sub-pathways and genera selected by any of sCCA and C-sCCA with newly determined tuning parameter pair. C-sCCA identifies 8 metabolism sub-pathways and 17 bacterial genera, while sCCA selects a broader set of 13 metabolic sub-pathways and 35 bacterial genera. By incorporating the compositional structure, C-sCCA achieves the comparable averaged CV value with a more focused selection of metabolic sub-pathways and bacterial genera.

Among the eight metabolic sub-pathways identified by C-sCCA, five belong to the *Lipid* super-pathway, two are part of the *Amino Acid* super-pathway, and only one, the *Advanced Glycation End-product*, is associated with the *Carbohydrate* super-pathway. For the bacterial genera, a comparison between C-sCCA results and the association analysis of bacterial genera and metabolic sub-pathways by Kim et al. (2020) reveals interesting patterns. The bacterial genera identified by C-sCCA are grouped into three clusters. The first two clusters predominantly show a negative association with the selected sub-pathways. In the first cluster, *Oscillospira*, *Clostridium*, *Ruminococcus*, *Adlercreutzia*, and *Dehalobacterium* are consistently selected by C-sCCA and were also reported in the findings of Kim et al. (2020). In the second cluster, *Odoribacter* is detected by C-sCCA but was not identified by Kim et al. (2020). In the third cluster, *Bacteroides*, *Eggerthella*, and *Butyrivibrio* stand out with the largest C-sCCA coefficients, showing strong positive correlations with the selected metabolic sub-pathways. In Kim et al. (2020), the genera were classified into two clusters, with the second cluster consisting of *Bacteroides*, *Epulopiscium*, and *Butyrivibrio*. Our hierarchical tree further reveals that *Epulopiscium* and *Eggerthella* exhibit very similar patterns, as they are grouped together.

## 6 Discussion

In this study, we developed a compositional sparse canonical correlation analysis (C-sCCA) framework for association analysis between microbiome data and other high-dimensional datasets, accounting for the compositional nature of microbiome sequencing data. We introduced two variants of the C-sCCA method: one for compositional vs non-compositional data, and another for compositional vs compositional data. Our results show that by incorporating the compositional constraint, we achieved improved selection of relevant taxa, enhancing both power and precision. Additionally, we extended our framework to incorporate prior structural information, such as the grouping of bacterial taxa, among the compositional components. Application of C-sCCA to real microbiome data demonstrated that it produced results that were biologically more interpretable.

There are several potential extensions to our work. While we primarily focused on the grouping structure of bacterial taxa, we could also exploit the hierarchical grouping structure (phylum to genus) and the phylognetic relationship by devising appropriate constraints on the weights w . For the hierarchical structure, we can derive a set of covariates each representing a hierarchical level, which can then be used to impose specific structures on the weights. Suppose for the j th taxa, we have a corresponding covariate $\xi_{j}$ . To incorporate such covariate information, we define the set of weights as

$$M_{\text{Covariate}}=\left\{w\in {\left[0,C_{U}\right]}^{p}:w_{j}=f\left(\xi_{j};\theta \right)\text{ for }\theta \in \Theta ,j\in \left\{1,2,\ldots ,p\right\}\right\},$$

where f(⋅;θ) is a prespecified class of functions parameterized by θ . We can also use the phylogenetic tree information by imposing a smoothness constraint, which depends on the pairwise patristic distances among the taxa. Suppose $d_{ij}$ is the patristic distance between the taxa i and j , we can consider the set

$$M_{\text{Smooth}}=\left\{w\in {\left[0,C_{U}\right]}^{p}:\underset{1\leq i<j\leq p}{\sum }\kappa \left(d_{ij}\right)|w_{i}-w_{j}|\leq \epsilon \right\},$$

for some decreasing function κ(⋅) of the distance and tuning parameter ϵ>0 .

We can also extend our approach to learn sub-spaces from multiple views, i.e., when we have multiple groups of measurements, $X_{\left(i\right)}\in R^{p_{i}}$ , for i=1,…,m on matching samples. This situation naturally arises in multi-omics studies, where methylomic, transcriptomic, metabolomic, and microbiome data are collected from a single group of individuals. A number of approaches for generalizing CCA to multiple views have been proposed in the literature, and some of these extensions are summarized in Kettenring (1971). We could adapt these multi-view methods to incorporate the compositional constraint.

There are several limitations to our framework. First, we did not simultaneously address the zero inflation commonly observed in microbiome data. We used a simple zero replacement strategy before running the C-sCCA. Although this strategy has been commonly used in microbiome data analysis at log scale (Zhou et al., 2022; Lin and Peddada, 2020), better methods can be developed such as replacing the log scale transformation by modified centered log-ratio transform designed for addressing zero inflation (Yoon et al., 2019), imposing another multinomial layer to account for sampling variability associated with the sequencing process (Chen and Li, 2013), or using more informative imputation methods such as mbDenoise (Zeng et al., 2022) and mbImpute (Jiang et al., 2021). Second, although our framework can select subsets of features that explain the largest correlation between the datasets, their detailed relationships can not be learned simultaneously. Developing methods that combine feature-level selection with the construction of feature-feature correlation networks is a promising area for future research. Third, we can enhance robustness to outliers through several strategies, including outlier detection and removal during data preprocessing, replacing empirical covariance estimators with robust estimators (Luo et al., 2024), and investigating the optimal choice of penalty functions (Chalise and Fridley, 2012), such as Huber loss, Tukey loss, or $L_{0}$ penalty (Lindenbaum et al., 2022). Fourth, while our work focuses on linear associations, future extensions of our composite sCCA framework could capture nonlinear relationships by integrating with kernel CCA (Akaho, 2001; Fukumizu et al., 2007), deep CCA (Andrew et al., 2013), or nonparametric CCA (Lancaster, 1958; Michaeli et al., 2016). Lastly, our framework assumes that the association is mediated through the ratios of the compositional components since only ratios are meaningful for compositional data. However, when the association is at the level of absolute abundance - where the total microbial load also matters - our method may not work well. This limitation is more inherent to the constraints of current sequencing technologies than to the method itself.

## Data Availability

Publicly available datasets were analyzed in this study. This data can be found here: Genotypes and Phenotypes (https://www.ncbi.nlm.nih.gov/gap) with the study accession number phs001204. v1. p1.
