# Supplementary material for: Structure-adaptive canonical correlation analysis for microbiome multi-omics data
Source: Front Genet. 2024 Nov 20;15:1489694. doi: 10.3389/fgene.2024.1489694 (PMC11626081; doi:10.3389/fgene.2024.1489694)
Supplement: Supplementary file 1 [file DataSheet1.pdf]

frontiers

Supplementary Material

SI PROOF

PROOF OF PROPOSITION 2.1. For the ease of notation, we set $\mathbf{h} = \widehat{\boldsymbol{\Sigma}}_{\mathbf{ZY}}\mathbf{a}^{(t)}$. Note that

$$\mathbf{b} = \begin{pmatrix} \mathbf{I}_{p-1} \\ -\mathbf{1}_{p-1}^\top \end{pmatrix} \mathbf{b}_{-p} := \mathbf{J}_p \mathbf{b}_{-p},$$

with $\mathbf{1}_{p-1}^\top = (1, 1, \ldots, 1) \in \mathbb{R}^{p-1}$. Set $\mathbf{K} = \mathbf{I}_{p-1} + \frac{1}{\sqrt{p}+1}\mathbf{1}\mathbf{1}^\top$ which satisfies that $\mathbf{K}^2 = \mathbf{J}_p^\top \mathbf{J}_p$. Define $l(\mathbf{c}, \lambda, \mathbf{w})$ as the solution to the following generalized Lasso (GLasso) problem (Tibshirani and Taylor, 2011)

$$l(\mathbf{c}, \lambda, \mathbf{w}) = \underset{\mathbf{b}_{-p} \in \mathbb{R}^{p-1}}{\arg\min} \frac{1}{2}\|\mathbf{K}^{-1}\mathbf{c} - \mathbf{K}\mathbf{b}_{-p}\|_2^2 + \lambda \|\mathbf{J}_p \mathbf{b}_{-p}\|_{1,\mathbf{w}}.$$

We can rewrite the optimization problem in (2) in the Lagrange form as

$$\min_{\mathbf{b}_{-p} \in \mathbb{R}^{p-1}} -\mathbf{h}^\top \mathbf{J}_p \mathbf{b}_{-p} + \lambda_{\mathbf{b}} \|\mathbf{J}_p \mathbf{b}_{-p}\|_{1,\mathbf{w}} + \tau(\|\mathbf{J}_p \mathbf{b}_{-p}\|_2^2 - 1)$$

for $\tau > 0$. Taking derivative with respect to $\mathbf{b}_{-p}$, and setting the derivative to be zero, we obtain

$$-\mathbf{J}_p^\top \mathbf{h} + \lambda_{\mathbf{b}} \mathbf{S}(\mathbf{b}_{-p}) + 2\tau \mathbf{K}^2 \mathbf{b}_{-p} = 0, \tag{S1}$$

where $\mathbf{S}(\mathbf{b}_{-p})$ denotes the sub-differential for $\|\mathbf{J}\mathbf{b}_{-p}\|_{1,\mathbf{w}}$. The Karush–Kuhn–Tucker conditions consist of (S1) together with $\tau(\|\mathbf{J}_p\mathbf{b}\|_2^2 - 1) = 0$. For $\tau > 0$, the condition $-\mathbf{J}_p^\top \mathbf{h} + \lambda_{\mathbf{b}} \mathbf{S}(\mathbf{b}_{-p}^{(t)}) + 2\tau \mathbf{K}^2 \mathbf{b}_{-p}^{(t)} = 0$ implies that

$$\mathbf{b}_{-p}^{(t)} = l\left(\frac{\mathbf{J}_p^\top \mathbf{h}}{2\tau}, \frac{\lambda_{\mathbf{b}}}{2\tau}, \mathbf{w}\right).$$

In this case, the constraint is binding and thus τ is chosen such that $\|\mathbf{J}_p \mathbf{b}_{-p}^{(t)}\|_2 = 1$. Notice that

$$\begin{aligned} l\left(\frac{\mathbf{J}_p^\top \mathbf{h}}{2\tau}, \frac{\lambda_{\mathbf{b}}}{2\tau}, \mathbf{w}\right) &= \underset{\mathbf{b}_{-p} \in \mathbb{R}^{p-1}}{\arg\min} \frac{1}{2}\left\|\mathbf{K}^{-1}\frac{\mathbf{J}_p^\top \mathbf{h}}{2\tau} - \mathbf{K}\mathbf{b}_{-p}\right\|_2^2 + \frac{\lambda_{\mathbf{b}}}{2\tau}\|\mathbf{J}_p \mathbf{b}_{-p}\|_{1,\mathbf{w}} \\ &= \underset{\mathbf{b}_{-p} \in \mathbb{R}^{p-1}}{\arg\min} \frac{1}{2}\left\|\mathbf{K}^{-1}\mathbf{J}_p^\top \mathbf{h} - 2\tau\mathbf{K}\mathbf{b}_{-p}\right\|_2^2 + \lambda_{\mathbf{b}}\|2\tau\mathbf{J}_p \mathbf{b}_{-p}\|_{1,\mathbf{w}} = \frac{1}{2\tau} l\left(\mathbf{J}_p^\top \mathbf{h}, \lambda_{\mathbf{b}}, \mathbf{w}\right). \end{aligned}$$

Thus we must have

$$\mathbf{b}_{-p}^{(t)} = \frac{l\left(\mathbf{J}_p^\top \mathbf{h}, \lambda_{\mathbf{b}}, \mathbf{w}\right)}{\|\mathbf{J}_p l\left(\mathbf{J}_p^\top \mathbf{h}, \lambda_{\mathbf{b}}, \mathbf{w}\right)\|_2}, \quad \mathbf{b}^{(t)} = \mathbf{J}_p \mathbf{b}_{-p}^{(t)}.$$

It is also not hard to verify that

$$\mathbf{J}_p l\left(\mathbf{J}_p^\top \mathbf{h}, \lambda_{\mathbf{b}}, \mathbf{w}\right) = g(\mathbf{h}, \lambda, \mathbf{w}).$$

If $\tau = 0$, the constraint is non-binding and we have $\mathbf{b}^{(t)} = \breve{\mathbf{b}}$. The conclusion thus follows.

REFERENCES

Tibshirani, R. J. and Taylor, J. (2011). The solution path of the generalized lasso. *The Annals of Statistics* 39, 1335–1371
